# Significance of active screening for detection of health problems in childhood cancer survivors

**DOI:** 10.3389/fped.2022.947646

**Published:** 2022-10-06

**Authors:** Yuri Yoshimoto-Suzuki, Daisuke Hasegawa, Yosuke Hosoya, Go Saito, Kyoko Nagase, Michiyo Gunji, Kyoko Kobayashi, Yasushi Ishida, Atsushi Manabe, Miwa Ozawa

**Affiliations:** ^1^Department of Pediatrics, St. Luke’s International Hospital, Tokyo, Japan; ^2^Course of Advanced and Specialized Medicine, Graduate School of Medicine, Juntendo University, Tokyo, Japan; ^3^Department of Nursing, St. Luke’s International Hospital, Tokyo, Japan; ^4^Department of Child & Family Health Nursing, St. Luke’s International University, Tokyo, Japan; ^5^Pediatric Medical Center, Ehime Prefectural Central Hospital, Ehime, Japan; ^6^Department of Pediatrics, Graduate School of Medicine, Hokkaido University, Hokkaido, Japan

**Keywords:** childhood cancer survivor, health problem, late effects, long-term care, screening

## Abstract

**Background:**

Childhood cancer survivors (CCSs) have a lifelong increased risk of chronic health problems, most of which are associated with the curative therapies. Recent studies have suggested that prospective active screening using comprehensive assessments for CCSs is superior in identifying undiagnosed chronic health problems.

**Methods:**

To assess the significance of active screening using comprehensive medical examinations for detecting chronic health problems in multiple organ systems in CCSs, we retrospectively compared the frequency and severity of health problems between two different cohorts of CCSs in a single institution: 110 CCSs who visited the outpatient clinic for regular follow-ups between December 2010 and December 2015 (regular follow-up group) vs. 58 CCSs who underwent comprehensive medical examinations between February 2016 and September 2019 (active screening group). CCSs were defined as patients aged ≥ 18 years who had been diagnosed as having childhood cancer ≥ 10 years before and had survived without cancer for ≥ 5 years.

**Results:**

Patient characteristics were similar between the two groups except for primary diagnosis (more brain tumors and embryonal tumors in the active screening group) and treatment history (more alkylating agents used and surgical interventions performed in the active screening group). The prevalence and the median number of health problems were significantly higher in the active screening group than in the regular follow-up group: 93% vs. 67% and 1.0 [0.0–8.0] vs. 2.0 [0.0–7.0] respectively. In term of organ-specific health problems, pulmonary dysfunction, neurocognitive impairment, ocular abnormalities, and dental abnormalities were identified more in the active screening group, partly because these problems had not been assessed in the regular follow-up group. Nevertheless, the prevalence of grade 3–5 health problems was similar between the two groups, except for pulmonary dysfunction.

**Conclusion:**

Active screening using comprehensive medical examinations was effective for identifying health problems in CCSs. Although the prevalence of severe problems identified by both approaches was similar, comprehensive medical examinations could detect overlooked problems such as severe pulmonary dysfunction, dental maldevelopment, and borderline intellectual functioning, which might have an impact on quality of life in CCSs.

## Introduction

Progress in the development of therapies for children with cancer has resulted in a >80% survival rate in developed countries, and children who overcome childhood cancer grow up as childhood cancer survivors (CCSs) (1). CCSs have a lifelong increased risk of chronic health problems, most of which are associated with the curative therapies. Chronic health problems in CCSs include cardiac dysfunction, pulmonary dysfunction, renal impairment, endocrine and reproductive disorders, growth impairment, neurocognitive impairment, and subsequent malignant neoplasms (2, 3). These health problems have previously been evaluated using self-report questionnaires or registry data. Recent studies have shown that prospective active screening using comprehensive systematic assessments for all CCSs was superior in identifying a substantial number of undiagnosed chronic health problems (4, 5). In fact, a large study of active screenings for adult CCSs revealed that the estimated cumulative prevalence of chronic health problems and serious/disabling or life-threatening problems at 45 years of age was 95.5 and 80.5%, respectively (6).

At St. Luke’s International Hospital, active screening for late-onset health problems among CCSs was started in February 2016. CCSs aged ≥ 18 years or older who were diagnosed as having childhood cancer at least 10 years before and remained in remission for ≥ 5 years underwent comprehensive medical examinations. Before the active screenings were initiated, patients treated for childhood cancer were evaluated for health problems during regular clinic visits at the discretion of the physician in accordance with the Children’s Oncology Group Long-term Follow-Up Guidelines (7) considering cancer type and treatment history.

In the present study, we compared the frequencies and severities of late-onset health problems between different approaches, namely, comprehensive medical examinations and regular clinic-based evaluations. The aim of this study was to assess the utility of active screening using comprehensive medical examinations in detecting chronic health problems of multiple organ systems in CCSs, which might be overlooked at regular clinic visits.

## Materials and methods

### Study design and participants

All data were obtained through the studies approved by the institutional review board of St. Luke’s International Hospital. In the present study, CCSs were defined as patients aged ≥ 18 years who were diagnosed with childhood cancer ≥ 10 years before and survived without cancer for ≥ 5 years. Data of 58 CCSs who underwent comprehensive medical examinations between February 2016 and September 2019 were prospectively collected (active screening group). We also reviewed the medical records of 147 CCSs who visited the outpatient clinic for regular follow-ups between December 2010 and December 2015, and their medical records were retrospectively analyzed. Of those 147 CCSs, 37 CCSs were excluded because they also underwent a comprehensive medical examination after February 2016, and were thus included in the active screening group in this analysis. Finally, 110 CCSs were assigned to the regular follow-up group. Written informed consent was obtained from all participants in the active screening group and opt-out consent involving provision of an information leaflet was obtained when the medical records of patients in the regular follow-up group were reviewed.

### Data collection

In the active screening group, participants underwent a comprehensive evaluation as follows: medical history, physical examination, resting blood pressure, complete blood cell count, comprehensive metabolic panel, fasting lipid profile, blood sugar, hemoglobin A_1_C level, endocrine–reproductive function (thyroid, gonadal, hypothalamic–pituitary axis function), urinalysis, fecal occult blood test, echocardiography, pulmonary function testing, audiological testing, ophthalmologic evaluation, dental evaluation, neurocognitive testing, bone mineral density testing, gynecological examination (female only), thyroid ultrasonography, abdominal ultrasonography, and brain MRI. These examinations were selected according to the comprehensive medical checkup system administered in healthy adults in Japan (8) and the St. Jude Lifetime Cohort (SJLIFE) study (6). In the regular follow-up group, medical records and the most recent examination results were reviewed, and information about health problems was extracted. Medical assessments for CCSs in the regular follow-up group were performed at the discretion of the pediatric oncologist in accordance with the guidelines (7) considering cancer type and treatment history. The cost of examinations for CCSs in the active screening group and the regular follow-up group was covered by the research grant and public health insurance, respectively. In both groups, cancer-related information, including type of cancer, cumulative doses of chemotherapy, information on hematopoietic cell transplantation, surgical interventions, and the dose and anatomical location of radiological therapy, was extracted from the medical chart for each patient.

### Definition of health problems and grading

The criteria for positive screening and the grading are shown in Table 1. Chronic health problems were classified according to the modified National Cancer Institute’s Common Terminology Criteria for Adverse Events (CTCAE) version 4.0, which was also utilized in the SJLIFE study (9). We made additional minor modifications to the SJLIFE-modified CTCAE as follows: obesity was defined as BMI ≥ 25 based on Asian criteria; HbA_1_c and cholesterol level was used in the evaluation of diabetes mellitus and hyperlipidemia, respectively, because the results of fasting tests were not always available in the regular follow-up group; hypothalamic–pituitary axis dysfunction was diagnosed using growth hormone provocation tests or ACTH (adrenocorticotropic hormone) stimulation tests in children in the regular follow-up group who were positive in screening for insulin-like growth factor-1 or morning cortisol level, respectively; hepatitis B or C virus infections were classified as grade 3 if they were serologically positive.

**TABLE 1:** Definition of health problems, screening tests, criteria for positive screening, and grading rubric.

Health problem	Screening test	Criteria for positive screening	Grading rubric (modified CTCAE v4.0)
**Cardiovascular**			
Cardiomyopathy or Heart valve disorders	Echocardiogram	EF < 50% Detection of presence of valvular sclerosis, stenosis, or calcifications or mild or greater regurgitation	EF 1: Not applicable 2: Resting EF < 50%–40% 3: Resting EF 39%–20% 4: Resting EF < 20%; refractory or poorly controlled heart failure due to drop in EF; on medical management; intervention 5: Death Heart valve disorder 1: Asymptomatic valvular thickening/calcifications with or without mild valvular regurgitation or stenosis by imaging 2: Asymptomatic; moderate regurgitation or stenosis by imaging 3: Symptomatic; severe regurgitation or stenosis by imaging; symptoms controlled with medical intervention 4: Life-threatening consequences; urgent intervention indicated 5: Death
**Cardiovascular risk factors**			
Hypertension	Blood pressure	BP ≥ 130/80 mmHg	1: Systolic BP 130–139 mm Hg or diastolic BP 80–89 mm Hg 2: Systolic BP 140–159 mm Hg or diastolic BP 90–99 mm Hg 3: Systolic BP ≥ 160 mm Hg or diastolic BP ≥ 100 mm Hg 4: Life-threatening consequences; urgent intervention indicated 5: Death
Dyslipidemia	Lipid panel	Total cholesterol ≥ 200 mg/dl, or LDL-C ≥ 140 mg/dl, or HDL-C < 40 mg/dl	1: Total cholesterol 200–300 mg/dl 2: Total cholesterol 300–400 mg/dl 3: Total cholesterol 400–500 mg/dl 4: Total cholesterol > 500 mg/dl 5: Not applicable
**BMI abnormalities**			
Obesity	BMI	BMI ≥ 25	1: Not applicable 2: BMI 25–29.9 kg/m^2^ 3: BMI 30–39.9 kg/m^2^ 4: BMI ≥ 40 kg/m^2^ 5: Not applicable
Underweight	BMI	BMI < 18.5	1: Not applicable 2: BMI < 18.5 kg/m^2^ 3: Not applicable 4: Not applicable 5: Not applicable
**Pulmonary**			
Abnormal pulmonary function	Pulmonary function tests	%VC < 80% or FEV1 < 80% predicted	%VC 1:%VC: 79–70% 2:%VC: 69–60% predicted 3:%VC: < 60% predicted 4: Not applicable 5: Not applicable FEV1 1: FEV1 (percentages of observed FEV1 related to its predicted value): 79–70% predicted 2: FEV1: 69–50% predicted 3: FEV1: 49–35% predicted 4: FEV1: < 35% predicted 5: Not applicable
**Metabolic**			
Liver dysfunction	ALT, AST	ALT ≥ 40 U/I AST ≥ 40 U/I	1: AST or ALT > ULN – 3.0 × ULN 2: AST or ALT > 3.0–5.0 × ULN 3: AST or ALT > 5.0–20.0 × ULN 4: AST or ALT > 20.0 × ULN 5: Not applicable
Kidney dysfunction	Creatinine, BUN, Urine analysis	eGFR < 60 ml/min/1.73 m^2^ ± Abnormal urinalysis	1: eGFR < LLN – 60 ml/min/1.73 m^2^ 2: eGFR 59–30 ml/min/1.73 m^2^ 3: eGFR 29–15 ml/min/1.73 m^2^ 4: eGFR < 15 ml/min/1.73 m^2^ 5: Death
**Endocrine or reproductive**			
HPA dysfunctions			
GH deficiency	GH provocation testing used to establish diagnosis in regular follow-up Active screening: IGF-1	GH dynamic testing: GH peak < criteria value Active screening: IGF-1 < -2SD	1: Not applicable 2: Asymptomatic; clinical or diagnostic observations only; intervention not indicated 3: Hormone replacement indicated or initiated 4: Severe symptoms; limiting self-care ADL; hospitalization indicated 5: Not applicable
ACTH deficiency	Low dose ACTH stimulation test used to establish diagnosis in regular follow-up Active screening: Morning cortisol	Low dose ACTH stimulation test: Cortisol < criteria value Active screening: Morning cortisol < 5 mcg/dL	
Diabetes mellitus	Serum glucose, HbA_1_c	HbA_1_C ≥ 6.4%	1: HbA_1_c 6.4–6.9% 2: HbA_1_c 7.0–7.9% 3: HbA_1_c > 8.0% 4: Life threatening consequences, urgent intervention indicated or initiated 5: Death
Primary hypothyroidism	Serum free T_4_, TSH	Both: Free T_4_ below normal range (<1.0 ng/dl) and TSH above normal range (≥4.0 IU/ml)	1: Not applicable 2: Asymptomatic; clinical or diagnostic observations only; intervention not indicated 3: Hormone replacement indicated or initiated 4: Severe symptoms; limiting self- care ADL; hospitalization indicated 5: Not applicable
Gonadal dysfunction			
Female	FSH, LH, estradiol	Amenorrhea before age 40 or estradiol < 17 pg/ml, FSH ≥ 30 mIU/ml	1: Not applicable 2: Asymptomatic; clinical or diagnostic observations only; intervention not indicated 3: Hormone replacement indicated or initiated 4: Severe symptoms; limiting self- care ADL; hospitalization indicated 5: Not applicable
Male	FSH, LH, testosterone	Receiving testosterone, and/or FSH > 10 mIU/ml, LH > 0.7 mIU/ml, Testosterone < 1.3 ng/ml	
**Neurocognitive**			
Neurocognitive impairment	WAIS	FSIQ < 85	1: FSIQ 85–90 2: FSIQ 70–88 3: FSIQ < 70 4: Not applicable 5: Not applicable
**Neurosensory**			
Ocular abnormalities	Ophthalmology consultation	Cataract, Intraocular pressure ≥ 21 mmHg, clinically significant abnormalities of retinal pigment, integrity, or vasculature, Low vision (corrected to 20/80 or worse)	1: Asymptomatic; clinical or diagnostic observations only; intervention not indicated 2: Symptomatic; moderate decrease in visual acuity (20/40 or better) 3: Symptomatic with marked decrease in visual acuity (worse than 20/40 but better than 20/200); operative intervention indicated (e.g., cataract surgery) 4: Blindness (20/200 or worse) in the affected eye 5: Not applicable
Hearing loss	Pure-tone audiometry	Measurable hearing loss	1: ≥ 40 dB at any frequency 6–12 kHz (Chang 1a); > 20 and < 40 dB at 4kHz (Chang 1b) 2: ≥ 40 dB at 4 kHz and above (Chang 2a); > 20 and < 40 dB at any frequency < 4kHz (Chang 2b) 3: ≥ 40 dB at 2 or 3 kHz and above 4: ≥ 40 dB at 1 kHz and above 5: Not applicable
**Skeletal**			
Osteoporosis	Dual-energy x-ray absorptiometry	BMD t-score ≤ -1.0	1: Radiologic evidence of osteoporosis or BMD t-score -1 to -2.5 (osteopenia) 2: BMD t-score < -2.5 3: Loss of height ≥ 2 cm 4: Not applicable 5: Not applicable
Dental abnormalities	Dental consultation	Missing teeth and/or microdontia and/or root change	1: Asymptomatic; hypoplasia of tooth or enamel 2: Impairment correctable with oral surgery 3: Maldevelopment with impairment not surgically correctable; disabling 4: Not applicable 5: Not applicable
**Infection**			
Hepatitis virus infection	Hepatitis B surface antigen and core antibody Hepatitis C antibody	Serologically positive	1: Not applicable 2: Not applicable 3: Present 4: Life-threatening 5: Death
**Cancer screening**			
Malignant neoplasms		Any malignant neoplasms	1: Not applicable 2: Not applicable 3: Present 4: Life-threatening 5: Death

ACTH, adrenocorticotropic hormone; ADL, activities of daily living; ALT, alanine aminotransferase; AST, aspartate aminotransferase; BP, blood pressure; BMD, bone mineral density; BMI, body mass index; BUN, blood urine nitrogen; CTCAE, Common Terminology Criteria for Adverse Events; eGFR, estimated glomerular filtration rate; FEV1, forced expiratory volume in the first second, FSH, follicle-stimulating hormone; FSIQ, Full scale intelligence quotient; GH, growth hormone; HbA_1_c, hemoglobin A_1_c; HDL-C, high-density lipoprotein cholesterol; HPA, hypothalamic-pituitary axis; IGF-1, insulin growth factor 1; LDL-C, low-density lipoprotein cholesterol; LH, luteinizing hormone; LLN, lower limit of normal; SD, standard deviation; TSH, thyroid-stimulating hormone; T4, thyroxine; VC; vital capacity; WAIS; ULN, upper limit of normal; Wechsler adult intelligence scale.

### Statistical analysis

Health problems were compared between the active screening group and the regular follow-up group. The significance of the presence and the number of health problems per CCS was tested by Fisher’s exact test, a non-parametric test, and one-way analysis of variance. Non-parametric variables were tested using the Mann–Whitney test and Kruskal–Wallis test. Data were analyzed using EZR (Saitama Medical Center, Jichi Medical University), which is a graphical user interface for R (The R Foundation for Statistical Computing, Vienna, Austria) (10); more precisely, it is a modified version of R commander, which is designed to perform statistical functions frequently used in biostatistics.

## Results

### Participant characteristics

The demographic, treatment, and diagnostic characteristics of the 110 CCSs in the regular follow-up group and the 58 CCSs in the active screening group are shown in Table 2. The proportion of males (43.6% vs. 46.6%), median age at diagnosis (6 years vs. 6 years), median age at the last follow-up (27.5 years vs. 25.5 years), and median duration of follow-up from diagnosis (19.0 years vs. 18.0 years) were not statistically different between the regular follow-up group and the active screening group. Hematological malignancies were more prevalent in the regular follow-up group than in the active screening group (81.8% vs. 70.7%), whereas the proportion of CNS tumors and embryonal tumors was higher in the active screening group. All CCSs received chemotherapy, but the proportion of CCSs receiving alkylating agents was higher in the active screening group. Nearly half of the CCSs in both groups received radiotherapy, and the main target organ was the brain. The proportion of CCSs who underwent surgical intervention was higher in the active screening group.

**TABLE 2:** Demographic, treatment, and diagnostic characteristics of CCSs in the regular follow-up group (*n* = 110) and the active screening group (*n* = 58).

Characteristics		Regular follow-up group	Active screening group	*P*-value
		(*n* = 110)	(*n* = 58)	
Sex	Male (%)	48 (43.6)	27 (46.6)	0.746
Primary diagnosis				0.025
	ALL	58 (52.7)	34 (58.6)	
	AML	13 (11.8)	3 (5.2)	
	Other hematological malignancies	10 (9.0)	1 (1.7)	
	Lymphoma	9 (8.2)	3 (5.2)	
	CNS tumors	3 (2.7)	5 (8.6)	
	Sarcoma	9 (8.2)	2 (3.4)	
	Embryonal tumors	6 (5.4)	10 (17.2)	
	Retinoblastoma	2 (1.8)	0 (0.0)	
Median age at diagnosis [years, range]		6 [0–19]	6 [0–16]	0.755
Median age at the last follow-up [years, range]		27.5 [18–49]	25.5 [18–42]	0.503
Median duration of follow-up [years, range]		19 [6–37]	18 [9–39]	0.413
Treatment exposure				
	Chemotherapy	110 (100)	58 (100)	
	Anthracyclines	71 (77.2)	44 (78.6)	1
	Alkylating agents	62 (67.4)	50 (89.3)	0.003
	Platinum	12 (13.0)	12 (21.4)	0.25
	Radiation	53 (48.2)	27 (46.6)	0.872
	Stem cell transplantation	18 (16.4)	9 (15.8)	1
	Surgery	19 (17.3)	20 (34.5)	0.02

ALL, acute lymphoblastic leukemia; AML, acute myeloid leukemia, CCSs, childhood cancer survivors; CNS, central nervous system.

### Prevalence of health problems in childhood cancer survivors

The prevalence of any health problems was significantly higher in the active screening group than in the regular follow-up group (93.1% vs. 67.3%; Table 3). In addition, the median number of any health problems was also higher in the active screening group than in the regular follow-up group (1.0 [0.0–8.0] vs. 2.0 [0.0–7.0]), and a third of CCSs in the active screening group had 4 or more health problems. In contrast, the prevalence and median number of severe health problems (≥ grade 3) were not significantly different between the two groups. Similar results were obtained from intrapatient comparisons in 37 CCSs who underwent regular follow-up and then active screening (data is not shown).

**TABLE 3:** Health problems in CCSs in regular follow-ups and active screening.

Health problems	Regular follow-up group (*n* = 110)	Active screening group (*n* = 58)	*P*-value
	No. of CCSs (%)	No. of CCSs (%)	
Presence of health problems			
No	36 (32.7)	4 (6.9)	<0.001
Yes	74 (67.3)	54 (93.1)	
Median [range] of problems per CCS	1.0 [0.0–8.0]	2.0 [0.0–7.0]	<0.001
No. of problems per CCS			
0	36 (32.7)	4 (6.9)	
1	25 (22.7)	16 (27.6)	
2	17 (15.5)	10 (17.2)	
3	15 (13.6)	9 (15.5)	
4	9 (8.2)	10 (17.2)	
≥ 5	8 (7.3)	9 (15.5)	
Presence of ≥ grade 3 health problems			
No	82 (74.5)	37 (63.8)	0.157
Yes	28 (25.5)	21 (36.2)	
No. of ≥ grade 3 health problems per CCS			
0	82 (74.5)	37 (63.8)	0.181
1	19 (17.3)	16 (27.6)	
2	10 (9.1)	5 (8.6)	
≥ 3	0 (0.0)	1 (1.7)	

CCS(s), childhood cancer survivor(s).

The median number of health problems was high in CCSs with CNS tumors (4.5 [1.0–8.0]; Table 4). Radiotherapy (2.5 vs. 1.0), hematopoietic cell transplantation (3.0 vs. 1.0), and platinum agents (3.0 vs. 1.0) were also associated with a higher number of health problems. The prevalence of severe health problems (≥grade 3) was also high in these CCSs: 62.5% in CCSs with CNS tumors, 41.2% in CCSs receiving radiotherapy, 51.9% in transplanted CCSs, and 50% in CCSs receiving platinum agents. The age at diagnosis, age at the last follow-up, and duration of follow-up were not associated with the mean number or severity of health problems.

**TABLE 4:** Prevalence of health problems in CCSs in regular follow-ups and active screening.

Health problems	Regular follow-up group (*n* = 110)			Active screening group (*n* = 58)			Regular vs. active all grades
			
	Any grades	Grade 3–4	Not tested	Any grade	Grade 3–4	Not tested	*P*-value
	*n* (%)	*n* (%)	*n* (%)	*n* (%)	*n* (%)	*n* (%)	
Cardiomyopathy or heart valve disorders	4 (3.6)	0 (0.0)	87 (79.1)	1 (1.7)	0 (0.0)	1 (1.7)	0.66
Hypertension	27 (24.5)	3 (2.7)	27 (24.5)	7 (12.1)	0 (0.0)	0 (0.0)	0.069
Dyslipidemia	40 (36.4)	0 (0.0)	6 (5.5)	25 (43.1)	0 (0.0)	0 (0.0)	0.409
BMI abnormalities							
Obesity	20 (18.2)	1 (0.9)	4 (3.6)	11 (19.0)	0 (0.0)	0 (0.0)	1
Underweight	15 (13.6)	0 (0.0)		13 (22.4)	0 (0.0)		0.281
Abnormal pulmonary function	3 (2.7)	1 (1.7)	104 (94.5)	12 (20.7)	4 (6.9)	0 (0.0)	<0.001
Liver dysfunction	9 (8.2)	0 (0.0)	0 (0.0)	6 (10.3)	0 (0.0)	0 (0.0)	0.777
Kidney dysfunction	6 (5.5)	3 (2.8)	1 (0.9)	0 (0.0)	0 (0.0)	0 (0.0)	0.094
HPA disorders	15 (13.6)	15 (13.6)	NA	10 (17.2)	5 (8.6)	1 (1.7)	0.649
Diabetes mellitus	3 (2.7)	0 (0.0)	17 (15.5)	1 (1.7)	1 (1.7)	0 (0.0)	1
Primary hypothyroidism	6 (6.5)	5 (4.5)	17 (15.5)	3 (5.2)	2 (3.4)	0 (0.0)	1
Gonadal dysfunction	20 (18.2)	10 (9.1)	20 (18.2)	7 (12.1)	5 (8.6)	0 (0.0)	0.38
Neurocognitive impairment	5 (4.5)	4 (3.6)	97 (88.2)	13 (22.4)	3 (5.2)	1 (1.7)	0.001
Ocular abnormalities	9 (8.2)	0 (0.0)	89 (80.9)	13 (22.4)	0 (0.0)	1 (1.7)	0.015
Hearing loss	3 (2.7)	0 (0.0)	100 (90.9)	6 (10.3)	0 (0.0)	0 (0.0)	0.065
Osteoporosis	3 (2.7)	0 (0.0)	103 (93.6)	3 (5.2)	0 (0.0)	0 (0.0)	0.417
Dental abnormalities	0 (0.0)	0 (0.0)	110 (100)	22 (37.9)	0 (0.0)	4 (6.8)	<0.001
Hepatitis B, Hepatitis C virus infection	7 (6.4)	7 (6.4)	22 (20.0)	5 (8.6)	5 (8.6)	0 (0.0)	0.754
Malignant neoplasms	5 (4.5)	5 (4.5)	NA	5 (8.6)	5 (8.6)	NA	0.316

BMI, body mass index; CCSs, childhood cancer survivors; HPA, hypothalamic-pituitary axis.

### Organ-specific health problems in childhood cancer survivors

Table 5 summarizes the prevalence of organ-specific health problems and the proportion of CCSs who did not undergo the screening tests for each health problem. Almost all the health problems were systematically evaluated in the active screening group by comprehensive medical examinations, whereas regular clinic-based evaluations failed to assess several organ-specific health problems, for example echocardiograms (the proportion of “not-tested” CCSs was 79.1%), pulmonary function tests (94.5%), neurocognitive evaluations (88.2%), ophthalmologic examinations (80.9%), audiometry (90.9%), bone mineral density tests (93.6%), and dental examinations (100%). These discrepancies in the frequency of organ-specific evaluations resulted in differences in the prevalence of relevant health problems between the regular follow-up group and the active screening group: pulmonary dysfunction (2.7% vs. 20.7%), neurocognitive impairment (4.5% vs. 22.4%), ocular abnormalities (8.2% vs. 22.4%), and dental abnormalities (unknown vs. 37.9%). However, the prevalence of severe organ-specific health problems (≥grade 3) was the same between the two groups except for pulmonary dysfunction (1.7% vs. 6.9%; *P* = 0.049). In fact, most of the ocular abnormalities reported in the active screening group were trivial (e.g., optic disk cupping, *n* = 11), while cataract was found in 2 CCSs. No CCSs in the regular follow-up group had dental examinations, whereas 54 of 58 CCSs (93.1%) in the active screening group underwent dental examinations and dental problems were reported in 22 CCSs: missing teeth in 14, microdontia in 8, and root change in 8. These dental abnormalities detected in the active screening group were not disabling and could be surgically corrected and were thus classified as grade 1–2. Of 12 patients who showed abnormal pulmonary function in active screening group, 11 had restrictive impairment and 4 were classified as grade 3–4.

**TABLE 5:** Health problems by clinical factors.

Clinical factors			CCSs with any health problems (%)	CCSs with ≥ grade 3 health problems (%)	Sum of health problems	
	
					Median [range]	*P*-value
Primary disease						
ALL	*n* = 92		72 (78.3)	21 (22.8)	2.0 [0.0–5.0]	0.002
AML	*n* = 16		11 (68.8)	6 (37.5)	2.0 [0.0–5.0]	
Other hematological malignancies	*n* = 11		9 (81.8)	3 (27.3)	1.0 [0.0–3.0]	
Lymphoma	*n* = 12		9 (75.0)	5 (41.7)	1.0 [0.0–6.0]	
CNS tumors	*n* = 8		8 (100.0)	5 (62.5)	4.5 [1.0–8.0]	
Sarcoma	*n* = 11		4 (36.4)	1 (9.1)	0.0 [0.0–2.0]	
Embryonal tumors	*n* = 16		13 (81.2)	6 (37.5)	2.5 [0.0–7.0]	
Retinoblastoma	*n* = 20		2 (100.0)	2 (100.0)	3.5 [3.0–4.0]	
Age at diagnosis (years)						
0–5	*n* = 75		56 (74.7)	28 (37.3)	2.0 [0.0–6.0]	0.951
5–10	*n* = 37		28 (75.7)	8 (21.6)	2.0 [0.0–7.0]	
≥ 10	*n* = 55		43 (78.2)	13 (23.6)	2.0 [0.0–8.0]	
Age at the last follow-up (years)						
18–20	*n* = 20		15 (75.0)	4 (20.0)	1.0 [0.0–6.0]	0.403
20–30	*n* = 89		65 (73.0)	24 (27.0)	2.0 [0.0–6.0]	
≥30	*n* = 59		48 (81.4)	21 (35.6)	2.0 [0.0–8.0]	
Duration of follow-up (years)						
<20	*n* = 88		64 (72.7)	18 (20.5)	1.5 [0.0–8.0]	0.489
20–30	n = 60		47 (78.3)	24 (40.0)	2.0 [0.0–7.0]	
≥30	n = 19		16 (84.2)	7 (36.8)	2.0 [0.0–6.0]	
Treatment exposure						
Chemotherapy						
Anthracyclines	Yes	*n* = 115	88 (76.5)	31 (27.0)	2.0 [0.0–7.0]	0.537
	No	*n* = 33	24 (72.7)	11 (33.3)	1.0 [0.0–8.0]	
Alkylating agents	Yes	*n* = 112	89 (79.5)	30 (26.8)	2.0 [0.0–8.0]	0.074
	No	*n* = 36	23 (63.9)	12 (33.3)	1.0 [0.0–6.0]	
Platinum	Yes	*n* = 24	20 (83.3)	12 (50.0)	3.0 [0.0–8.0]	0.007
	No	*n* = 124	92 (74.2)	30 (24.2)	1.0 [0.0–5.0]	
Radiation	Yes	*n* = 80	69 (86.2)	33 (41.2)	2.5 [0.0–8.0]	< 0.001
	No	*n* = 88	59 (67.0)	16 (18.2)	1.0 [0.0–6.0]	
Stem cell transplantation	Yes	*n* = 27	24 (88.9)	14 (51.9)	3.0 [0.0–7.0]	0.017
	No	*n* = 140	103 (73.6)	34 (24.3)	1.0 [0.0–8.0]	
Surgery	Yes	*n* = 39	31 (79.5)	16 (41.0)	2.0 [0.0–8.0]	0.209
	No	*n* = 129	97 (75.2)	33 (25.6)	2.0 [0.0–6.0]	

ALL, acute lymphoblastic leukemia; AML, acute myeloid leukemia, CCSs, childhood cancer survivors; CNS, central nervous system.

## Discussion

This report delineates the prevalence and severity of health problems across multiple organ systems in adult CCSs, as assessed by comprehensive medical examinations and regular clinic-based evaluations performed at a single center in Japan. Similar to previous reports, the incidence and severity of chronic health problems were associated with specific clinical factors such as CNS tumors, radiotherapy, hematopoietic cell transplantation, and platinum agents.

Active screening successfully identified more health problems compared with regular clinic-based evaluations (93.1% vs. 67.3%). This result might be attributable to efficient screening using comprehensive examinations to evaluate systemic health problems. In fact, several examinations were rarely performed in CCSs at regular follow-ups: echocardiograms, pulmonary function tests, neurocognitive evaluations, ophthalmologic examinations, audiometry, bone mineral density tests, and dental examination. Active screening revealed that pulmonary dysfunction, neurocognitive impairment, ocular abnormalities, and dental abnormalities were more prevalent than previously thought. In the SJLIFE study, prospective and risk-based systematic screening of health problems among CCSs revealed that the prevalence of newly diagnosed neurocognitive and neurosensory deficits, heart valve disorders, and pulmonary dysfunction was particularly increased, which was almost similar to our study. The examinations administered in the active screening group were selected according to the comprehensive medical checkup system administered in healthy adults in Japan (8) and the SJLIFE study (6). Comprehensive medical checkups allow for early detection of diseases in healthy adults and are widely used in Japan. Considering that this medical program is not covered by public health insurance, the cost-effectiveness of comprehensive medical examinations for CCSs needs to be further evaluated.

Most of the problems overlooked in the regular follow-up group were classified as grade 1–2; however, this may not mean that active screening resulted in overdiagnosis of clinically insignificant health problems in CCSs. For example, grade 1–2 dental anomalies were found in 40% of CCSs in the active screening group. Although these dental problems were not disabling and could be surgically corrected, they had potential impact on functional and aesthetic prognoses. Previous studies showed that the prevalence of root abnormalities ranged from 1.3 to 5.6%, and microdontia from 1 to 2% in a population of healthy children (11). The prevalence of hypodontia varies between 4 and 8% depending on the ethnic background (12). Pulmonary dysfunction and neurocognitive impairment were also more prevalent in the active screening group. Considering that the morbidity rate of pulmonary dysfunction was equivalent to that in the SJLIFE study (6) and that 4 of 12 pulmonary dysfunctions found in the active screening group were classified as ≥ grade 3, active screening successfully identified severe underlying pulmonary dysfunctions. Careful follow-up, education, and appropriate intervention such as pneumococcal vaccination should be implemented for CCSs with asymptomatic pulmonary dysfunction to prevent future worsening of pulmonary function. Identification of borderline intellectual functioning in a substantial number of CCSs in the active screening group is also important because of the potential impacts on quality of life. Further investigation is needed to clarify the significance of early interventions in CCSs with borderline intellectual functioning detected by active screening. The significance of ophthalmological abnormalities found in the present study may be controversial. Eleven of the 13 ophthalmological abnormalities in the active screening group were optic disk cupping, which were asymptomatic and concluded to be a non-specific finding by a more detailed follow-up. Because ocular abnormalities affect quality of life in CCSs, further investigation and analysis are needed to determine the importance of early detection of subtle changes in ophthalmological findings.

Because the prevalence of obesity, hypertension, and several laboratory abnormalities such as dyslipidemia and endocrinopathy did not increase by active screening, routine physical examinations and blood tests were adequate to identify these problems. Despite the low examination rate in the regular follow-up group, the similar prevalence of cardiac dysfunction and osteoporosis between the two groups was surprising. One plausible explanation may be that these problems were appropriately evaluated based on treatment history in the regular follow-up group. Another explanation may be that the follow-up duration of the present study was too short to detect subclinical cardiotoxicity among asymptomatic CCSs with normal ejection fractions. Cardiac dysfunction increases markedly with aging, and as many as 1 in 8 of CCSs treated with anthracyclines and chest radiation therapy will have a life-threatening cardiovascular event within 30 years after treatment (13). Further follow-up using parameters for early detection of subclinical diastolic dysfunction, including strain measurements by speckle tracking, may be required (14).

To our knowledge, this is the first study to prospectively explore systemic health problems in Asian CCSs using comprehensive screening for multiple organs (15). Most previous studies of CCSs in Asian countries have focused on epidemiological research or organ-specific toxicities (15). Health problems in CCSs are affected by a complex of multiple factors. Differences in genetic variations, socioeconomic status, health behaviors, lifestyle, treatment regimens, and clinical practice can be reflected in the prevalence of health problems in CCSs. The relatively low incidence of obesity in our cohort may exemplify the ethnic difference. Differences in genetic variations that influence the incidence of chronic health problems in CCSs between Asian and other populations should be explored in the future.

Several limitations should be considered when interpreting the results of this study. The major limitation is the small sample size. Second, the follow-up period was short, and the participants were relatively young. The health problems of CCSs are known to increase with age (16). Although the number and severity of health problems did not increase with age and from the time since diagnosis of pediatric cancer in this study, continuing active screening is expected to contribute to the prompt identification of health problems in CCSs. In addition, generalizability of our findings may be skeptical because most long-term follow-up clinics cannot afford to do the comprehensive medical testing. We believe that one of the significances of our study is that active screening could identify several overlooked problems, which was not regarded as severe based on CTCAE-based criteria but may affect quality of life in CCSs as described above. We are now planning detailed analyses on these subclinical but important health problems and will propose refined risk-based regular follow-up program including pulmonary function tests, neurocognitive evaluations, and dental examination.

## Conclusion

Our study revealed that active screening using comprehensive medical examinations can identify health problems in CCSs efficiently. Although the prevalence of severe problems was the same between regular clinic-based evaluations based on follow-up guidelines and comprehensive medical examinations, the latter detected several overlooked problems such as pulmonary dysfunction, dental maldevelopment, and borderline intellectual functioning, which might have an impact on quality of life in CCSs. The cost-effectiveness of active screening should be further addressed in future studies.

## Data availability statement

The original contributions presented in this study are included in the article/supplementary material, further inquiries can be directed to the corresponding author.

## Ethics statement

Written informed consent was obtained from all participants in the Active screening group and opt-out consent involving provision of an information leaflet was obtained when the medical records of patients in the Regular follow-up group were reviewed. This study was approved by the Ethics board of St. Luke’s International Hospital.

## Author contributions

YY-S, DH, YH, KK, YI, AM, and MO conceived and designed the study. GS, KN, and MG collected the clinical information and data. YY-S and DH analyzed the data, interpreted the results, and wrote the manuscript. YH, YI, AM, and MO contributed to the interpretation of the data and revised the manuscript. All the authors reviewed the manuscript and approved the final version of the manuscript.
